# A case of recurrent herpes simplex 2 encephalitis, VZV reactivations, and dominant partial interferon-gamma-receptor-1 deficiency supports relevance of IFNgamma for antiviral defense in humans

**DOI:** 10.1186/s40348-020-00106-4

**Published:** 2020-10-14

**Authors:** Julia Körholz, Nicole Richter, Jochen Schäfer, Catharina Schuetz, Joachim Roesler

**Affiliations:** 1 Children’s Department, Immunology, University Hospital Dresden, Fetscherstraße 74, 01307 Dresden, Germany; 2 Department of Neurology, University Hospital Dresden, Fetscherstraße 74, 01307 Dresden, Germany

**Keywords:** IFNγR-deficiency, Viral infections, HSV2-meningoencephalitis

## Abstract

**Background:**

Unlike infections with mycobacteria, reports of unusual viral infections in interferon-gamma-receptor (IFNγR) deficient patients are scarce. Therefore, discussion about increased susceptibility to viral infections in these patients is ongoing.

**Case presentation:**

We describe a 51-year-old male with dominant partial interferon-gamma-receptor-1 (IFNγR1)-deficiency and recurrent Herpes simplex 2 meningoencephalitis as well as other viral reactivations since childhood.

**Conclusions:**

This case further confirms an enhanced risk for viral disease in IFNγR-deficient patients and a role of interferon gamma for human antiviral defense.

## Background

Increased susceptibility to mycobacterial infection is the main feature of IFNγR deficiency [5]. In addition, some authors also report viral infections. However, documented cases are scarce [6, 16] and the notion of increased susceptibility to viral infections in IFNγR-deficiency therefore remains unsettled. Here, we describe an IFNγR deficient patient with severe and recurrent infections with herpes viruses.

The herpes virus family comprises over 100 different viruses, which are classified according to the architecture of their virion. They all share the ability to persist in their hosts as a latent infection and to reactivate in case of (transient and/or local) immunodeficiency [17]. Eight herpes viruses have been isolated from humans. Particularly in immunocompromised individuals, herpes viridae can cause severe infections [10]. In a large study from Finland, it was found that of all diagnosed viral encephalitides 29% were caused by varicella zoster virus (VZV) and 11% by Herpes simplex virus (HSV). HSV infection can have a severe clinical course causing neurologic damage [13, 15]. HSV2 infections of the CNS are far less frequently diagnosed than HSV1-infections because HSV2 mainly causes a sexually transmitted [1] local genital infection. The worldwide prevalence of HSV2 is estimated to be 11.3% with large regional differences (7.5% in Europe, 32% in Africa) [14].

Type I and III interferons are known to play a critical role in our innate immune system’s virus defense. They are produced in response to viral infections, activate a JAK/STAT-pathway and thereby induce the expression of interferon stimulated genes (ISG). The products of ISG then mediate the antiviral effect [9]. The role of IFNγ in human viral defense is less clear and many patients with IFNγR-deficiency can successfully cope with such infections.

Patients with IFNγR1-deficiency are not only prone to systemic infections with mostly non-tuberculous mycobacteria, but also Salmonella, Listeria, and others [5, 18]. Two forms of IFNγR1-deficiency can be differentiated: complete deficiency is inherited in an autosomal recessive manner and those patients suffer from severe and life-threatening infections while patients with dominantly inherited partial receptor deficiency often show a much milder course of their disease [5, 7].

So far, three patients of different families with autosomal-recessive complete IFNG-R1-deficiency and unusual viral infections have been described [6]. In two more cases, a possible relation between herpes-associated malignancy and complete autosomal-recessive IFNGR1-deficiency was found [2, 3]. Furthermore, three patients with enhanced susceptibility to viral infections from two different families with autosomal-dominant partial IFNG-R1-deficiency have been reported [6, 16].

This is the first report of such a patient with recurrent HSV2 encephalitis.

## Case presentation

A 51-year-old male patient who had been in good general condition over the last 2 years was admitted to our hospital with severe headaches, a feeling of weakness in his legs, noise sensitivity and nausea. An MRI revealed signs of chronic intracranial pressure with hydrocephalus (extended ventricles and diapedesis of CSF). The ophthalmologic examination revealed bilateral papilloedema. In his ventricular CSF, HSV2 was detected by PCR (3 × 10^4^ copies/mL, ref. < 500; in CSF: protein 1130mg/L, ref. < 150; cell count 221MPt/L, ref. < 2) and a renewed HSV2 meningoencephalitis was diagnosed like 2 years ago. With external ventricular drainage and i.v. administration of aciclovir (1950 mg/day for 21 days) the patient improved. The intraventricular drain could be removed and the hydrocephalus did not recur.

The patient’s medical history reached back to early childhood. On day 4 after birth, he received a BCG vaccination under immunoglobulin substitution. At that time, physicians assumed that this substitution could provide protection, in case the patient had inherited a primary immunodeficiency. They suspected a primary immunodeficiency, because his older sister had suffered from generalized BCGitis after vaccination. Despite IVIG substitution, the boy developed BCGitis with lymphadenitis axillaris and fever, and a generalized exanthema a few days later. M. bovis (type BCG) was detected in granulomas from a lymph node biopsy and the boy was treated with INH and streptomycine. At the age of 4 months, the patient developed alleged hepatitis tuberculosa (multiple granulomas with caseating necrosis in a liver biopsy, no microbiological evidence of mycobacteria) and was therefore started on another anti-tuberculous medication. A few weeks later, after an episode of enteral salmonellosis, he developed high fever and right leg pain and was diagnosed with osteomyelitis of the right tibia caused by Salmonella typhimurium. At the age of 5 years, the patient developed VZV pneumonia during his first chickenpox infection. This episode is well documented because he was an inpatient for two weeks. At age 6, he was operated for presumed appendicitis. Retrospectively, pronounced lymphadenitis mesenterialis due to Yersinia pseudotuberculosis had mimicked appendicitis. At age 31, the patient suffered from severe chickenpox with a generalized pronounced vesicular rash and had another episode of VZV pneumonia requiring hospital admission at the same time as his son fell ill with chickenpox. During the following 15 years, the patient had repeated episodes of thoracic shingles (typical painful segmental vesicular rash, diagnosed by general practitioner, treated with oral and local aciclovir administration), which resolved without sequelae, but he was otherwise healthy. At age 49, he was admitted to our hospital with personality change and somnolence and was finally diagnosed with meningoencephalitis. HSV2 was detected in CSF by PCR (9 × 10^6^ copies/mL, protein 3460 mg/L, cell count 1617 MPt/L in CSF). The MRI showed mild signs of inflammation. The meningoencephalitis was treated successfully with i.v.-aciclovir (2250 mg/day for 14 days). In the following 2 years, the patient reported repeated and sometimes severe headaches some of which he treated with aciclovir on his own without consulting a physician. This patient had been reported by Jouanguy et al. [11] and Dorman et al. [5] (Table 1 nr. 31C) before the onset of the remarkable viral infections.

The family history of the patient revealed that his mother had suffered from alleged tuberculosis of the urinary bladder early in life, which was cured by anti-tuberculous therapy. She died at age 30 from chronic progressive hepatitis that could not be further specified.

The patient’s sister developed generalized BCGitis after vaccination and was treated with anti-tuberculous therapy. A relapse could be controlled with tuberculostatic treatment, and no other infectious complications occurred during childhood. However, the sister died at age 27 from an intestinal mycobacterial infection, which had been misinterpreted as Crohn’s disease and therefore treated with steroids.

The patient described in this report has two sons with the same IFNγR1-deficiency. The older son remained clinically healthy. The younger son suffered from various infections such as VZV-pneumonia, VZV-meningoencephalitis, shingles, suspected HHV-6 hepatitis, lymphadenitis mesenterialis, and mycobacterial osteomyelitis [16]. The patient’s daughter and a nephew are not affected. Genetic testing of the patient and his sons revealed the common c.818–821del4 mutation in one allele of the *IFNGR1*-gene causing dominant partial IFNγR1 deficiency. Whole exome sequencing (WES) of the patient, his wife and the clinically affected son did not reveal any potential causative variation other than IFNɣR1 deficiency. Jouanguy et al. recently published inborn errors of human antiviral immunity to herpes viruses [10]. All genes listed by Jouanguy et al. (Table 1 [10]) were unaffected as determined by WES.

## Discussion and conclusions

Previous reports of viral infections in human IFNγR-deficiency are scarce [6, 16]. After the first report [6], Casanova cautioned not to interpret the findings prematurely as clear evidence for increased susceptibility to viral infections in this group of disorders [4]: severe mycobacterial illness could have led to additional weakness of defense mechanisms. Furthermore, most episodes of viral infections except for two remained localized. All patients responded well to treatment, and none had a relapse. In addition, many IFNγR-deficient patients had normal antibody-titers against viruses without severe respective viral infections in their medical history.

However, the disease course in our two patients reported here and previously [16] further support the notion that IFNγ signaling is of some importance in human defense against viruses. Neither of the two patients had any concurrent illnesses at the time of or shortly before the viral infections. These infections did not remain localized, but also involved the CNS. They should not be regarded as mild because only aciclovir treatment may have prevented organ damage. Furthermore, the viral infections recurred, most probably due to reactivation. In addition, two different herpes viruses, VZV and HSV2, occurred in our patient and isolated HSV2-encephalitis is rare.

In summary, the clinical course of the viral infections in this patient and in his son [16] were unusual, even though similar courses have also rarely been reported in patients without obvious immunodeficiency. This had, however, not been completely ruled out in the latter reports [8, 19, 20].

Including this report, six IFNγR-deficient patients from five families falling ill with relevant viral infections have so far been described (3 patients from 2 families with partial deficiency and 3 patients from 3 families with complete deficiency). Since five families with different origins are involved, there is diversity in the genetic background. Therefore, it is unlikely that one unknown genetic variant in the background alone causes susceptibility to viral infections. Furthermore, in our family, no additional causative genetic defects have been found by WES. Nevertheless, other (environmental or genetic) factors must play an additional role. Variants not revealed by WES in any of the many genes involved in viral defense that lower anti-viral efficacy (or a combination of such variants) are plausible additional factors. This is a necessary conclusion because most patients with IFNγR-deficiency can fight viruses normally.

In two different cases (an HHV8-associated Kaposi-sarcoma and an EBV related lymphoma), a possible relation between viral disease and complete IFNγR-deficiency has been described as well [2, 3]. However, in these cases, a decreased anti-malignant rather than a decreased anti-viral effect may perhaps have been prominent. Many more patients with IFNγR-deficiency have been reported who developed mycobacterial and sometimes salmonella infections. Viral infections related to IFNγR-deficiency therefore remain rare. The reason is probably effective redundancy in viral defenses.

Murine disease models and murine and human cell lines reveal possible antiviral mechanisms mediated by IFNγ in humans [12]. IFNγ inhibits viral entry at both extracellular and intracellular stages: replication by disrupting replication niche, gene expression by hindering translation, stability by impeding nucleocapsid assembly, release by breaking the disulfide bond of a necessary cellular interaction partner, and reactivation by suppressing the transcription of a viral master regulator.

To decide, which of these mechanisms are essential for healthy individuals or for patients needs further investigations. The viral infections in our patients support the notion that at least some of these mechanisms may be relevant. Therefore, life-vaccinations against herpesviridae (VZV) are potentially risky in such patients.

In summary, this and our previous reports reveal that in some patients with IFNγR-deficiency, viruses cause severe infections.

## Data Availability

The datasets used and analyzed during the current study are available from the corresponding author on reasonable request.

## Abbreviations

CNS: Central nervous system
CSF: Cerebrospinal fluid
IFNγR: Interferon-gamma-receptor
INH: Isonicotinic acid hydracide
